# The National Association for Supplying Female Medical Aid to the Women of India

**Published:** 1901-08-10

**Authors:** 


### THE NATIONAL ASSOCIATION FOR SUPPLYING FEMALE MEDICAL AID TO THE WOMEN OF INDIA.

The report of this Association—better known as "The Countess of Dufferin's Fund"—for the year 1900 is eminently satisfactory, showing, as it does, the steady progress which is being made on all sides to provide the women of India of every class with efficient medical assistance. There are now 247 hospitals, wards, and dispensaries of various kinds for the treatment of females which are officered by women, and most of them governed by, or affiliated to, the Association, though some are now independent of it. Also in the financial position of the institution a marked improvement is visible—an improvement which has only recently commenced, and the full benefit of which is likely to be yet more apparent in the next report than it is in the present. The encouraging progress in Bombay is entirely due to Lady Northcote's personal example and energy; in Madras Lady Ampthill has evinced a keen interest in the work, and made a most successful appeal to the public; the outlook in the North-Western Provinces, mainly owing to the liberality of the Maharaja of Bubrampur has substantially improved the financial status of the Provincial Branch; whilst the prospect in Bengal is not discouraging. But the greatest need of the Fund is further inducements to women to train as native midwives to work in the zenanas. The late Queen Victoria deeply realised this, and when Lady Curzon wrote to her on the subject about two years before her death, her Majesty at once telegraphed back a hope that it might be found feasible to promote the plan which was then being discussed. In order to carry this out some sort of endowment or scholarship for the training of midwives of good caste must be established. For this to be done on a sufficient scale a large sum of money will be required, and Lady Curzon suggested at the last meeting over which she presided that possibly some persons might be willing to take this means of commemorating her reign, and thus do much to relieve those who, like herself, are women and mothers. Already there are 16 female hospitals in India called Victoria after the late Empress, and many women showed real grief at the sad news of last January. Therefore it seems very fitting that a memorial of the good Queen should be one who should bring increased health and happiness to the women of her Empire. It has always been found very difficult to persuade the women of India to leave their homes, and this obstacle to aiding them in their distress would be overcome in a large measure if there were trained midwives to practise in outlying places. Meanwhile in some districts there is no doubt that *purdah* women are beginning to flock readily to female hospitals. In the Victoria Jubilee Hospital at Quilon they have lately been very apparent, and at Hyderabad (Deccan) out of the patients treated 507 were strictly *purdah* women of the better classes. It is felt that better *purdah* arrangements would lead to increased attendance on the part of *purdah-neshin* women. At Hyderabad (Sind) there were 609 *purdah-neshin* ladies attending the out-patient department in 1900, as against 201 the year before, and the in-patients admitted were 14, as against 4 in the previous twelve months. The wonder is, not that so comparatively little success has been obtained, but rather that so much has been accomplished, when, even nowadays in a city like Calcutta, it is understood that many women still consider it a disgrace to enter a *purdah* hospital; while in Rajputana others deem themselves defiled and proceed to bathe if they shake hands with an English lady. In the face of all their prejudice it is good to read that in Bombay there is an increase of 12,000, and in Madras an increase of 49,000, patients over the numbers treated last year. In the Victoria Caste and Gosha Hospital, which is open only to women of the *purdah* class, 18 of the 64 beds have been permanently endowed by native gentlemen. Again, during the year, the Maharaja of Nepa set aside a building to be reserved as a hospital for women only. Miss Yamini Sen, L.M.S., who was formerly a student of the Calcutta Medical College, was selected by the Durbar as the lady doctor in charge, and all women in poor circumstances are able to obtain there gratuitous advice and treatment. The Durbar subscribed freely towards the cost of the hospital, which is conducted on the latest scientific principles. Further details are not at present obtainable, but the circumstance itself is most encouraging to those who are anxious to help the women of India. A summary of the work of the Countess of Dufferin's fund shows that either in hospital or at their own homes 1,667,329 women and children have received surgical or medical benefit during 1900; that 37 lady doctors of the first grade, 80 assistant surgeons, and 290 hospital assistants and practitioners of the third grade are employed in the various zenana hospitals and institutions; that, including nurses and compounders, 379 women are at present studying medicine, etc., at the various classes and that the total receipts up to date amounted to 11,86,140 rs. As might have been expected, owing to many causes, the amount of subscriptions from the United Kingdom fell from £656 in 1889 to £325 in 1900, but this is probably only a temporary decrease.

